# Supplementary material for: Study on the Concentration of Acrylic Acid and Acetic Acid by Reverse Osmosis
Source: Membranes (Basel). 2020 Jul 6;10(7):142. doi: 10.3390/membranes10070142 (PMC7408086; doi:10.3390/membranes10070142)
Supplement: Supplementary file 1 [file membranes-10-00142-s001.pdf]

Cover Page for Supplementary Materials

Manuscript title:

Study on the concentration of acrylic acid and acetic acid by reverse osmosis

Author list:

Qian Liu [1,2], Lixin Xie [1,2,*] , Hanxiao Du [1,2], Shichang Xu [1,2], and Yawei Du [3,*]

[1] School of Chemical Engineering and Technology, Tianjin University, Tianjin 300072, P. R. China;

[2] Tianjin Key Laboratory of Membrane Science and Desalination Technology, State Key Laboratory of Chemical Engineering (Tianjin University), Tianjin 300072, P. R. China;

[3] School of Chemical Engineering and Technology, Hebei University of Technology, Tianjin 300130, P. R. China;

*Correspondence: xie_lixin@tju.edu.cn (L.X.X.); sonicduyawei001@126.com (Y.W.D);

Total number of pages (including the cover page): 8 pages

List of Tables and Figures:

Page 3 **Figure S1 Determination of the water permeability of the membranes for the acid solution.**

Page 4 **Figure S2 Determination of the acid transport coefficients of the C5 (a) and C6 (b) by fitting the acid retention and water flux at different pressures.**

Page 5 **Table S1. Comparison of the acetic retention with those in the literature.**

Page 6 **Figure S3 AFM images of RO membranes: (a, d) prior to exposure and (b, c, e, f) after 6 months of exposure to the acid solutions.**

Page 7 **Figure S4. The C1s spectra and N1s spectra of C5 membranes: (a, b) prior to exposure and (c, d, e, f) after 6 months of exposure to**

the acid solutions.

Page 8 **Figure S5 The C1s spectra and N1s spectra of C6 membranes: (a, b) prior to exposure and (c, d, e, f) after 6 months of exposure to the acid solutions.**

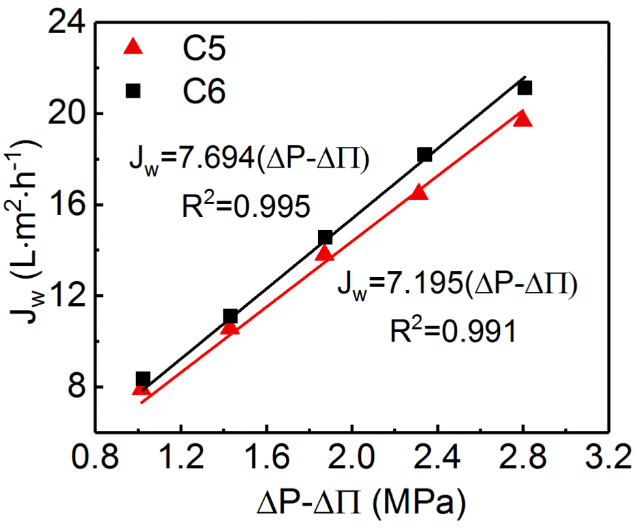

Figure S1. Determination of the water permeability of the membranes for the acid solution.

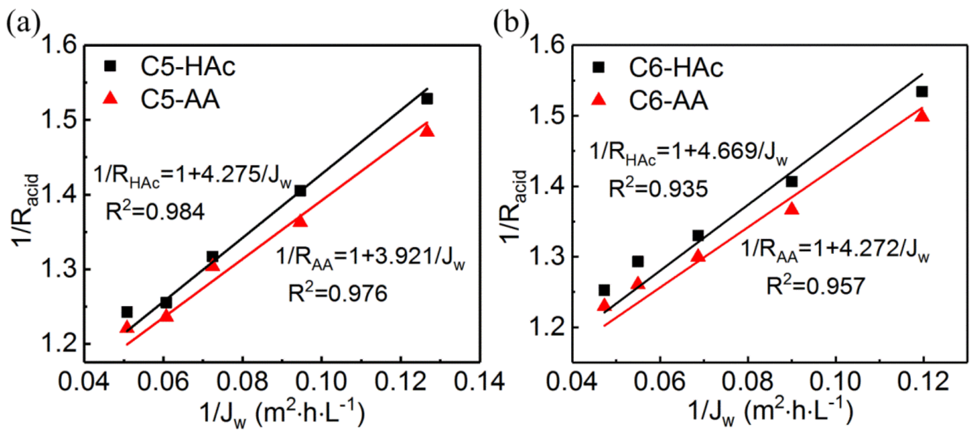

Figure S2. Determination of the acid transport coefficients of the C5 (a) and C6 (b) by fitting the acid retention and water flux at different pressures.

Table S1. Comparison of the acetic retention with those in the literature.

Membrane	Pressure (MPa)	pH	Feed solution	HAc retention (%)	Ref.
AG	4.0	-	7% HAc+15% glucose	< 40	[27]
RO98pHt	4.0	2.93	25 g/L xylose+10g/L glucose+ 5 g/L HAc	65.94	[20]
BW30FR	4.0	2.78	model solution	78.72	[29]
XLE	3.0	3	model solution	80	[30]
C5	4.0	2.39	2.5% AA+1.5% HAc	81.92	This work
C6	4.0	2.39	2.5% AA+1.5% HAc	81.32	

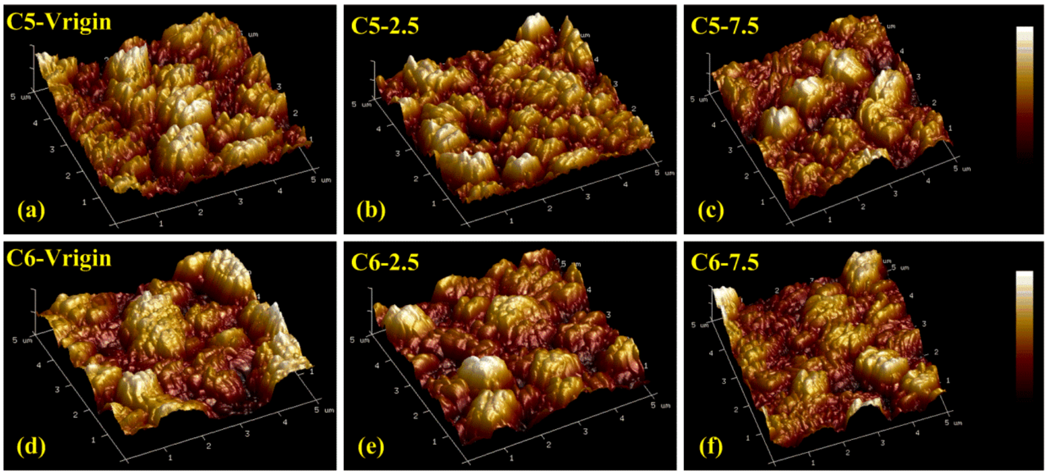

Figure S3. AFM images of RO membranes: (a, d) prior to exposure and (b, c, e, f) after 6 months of exposure to the acid solutions.

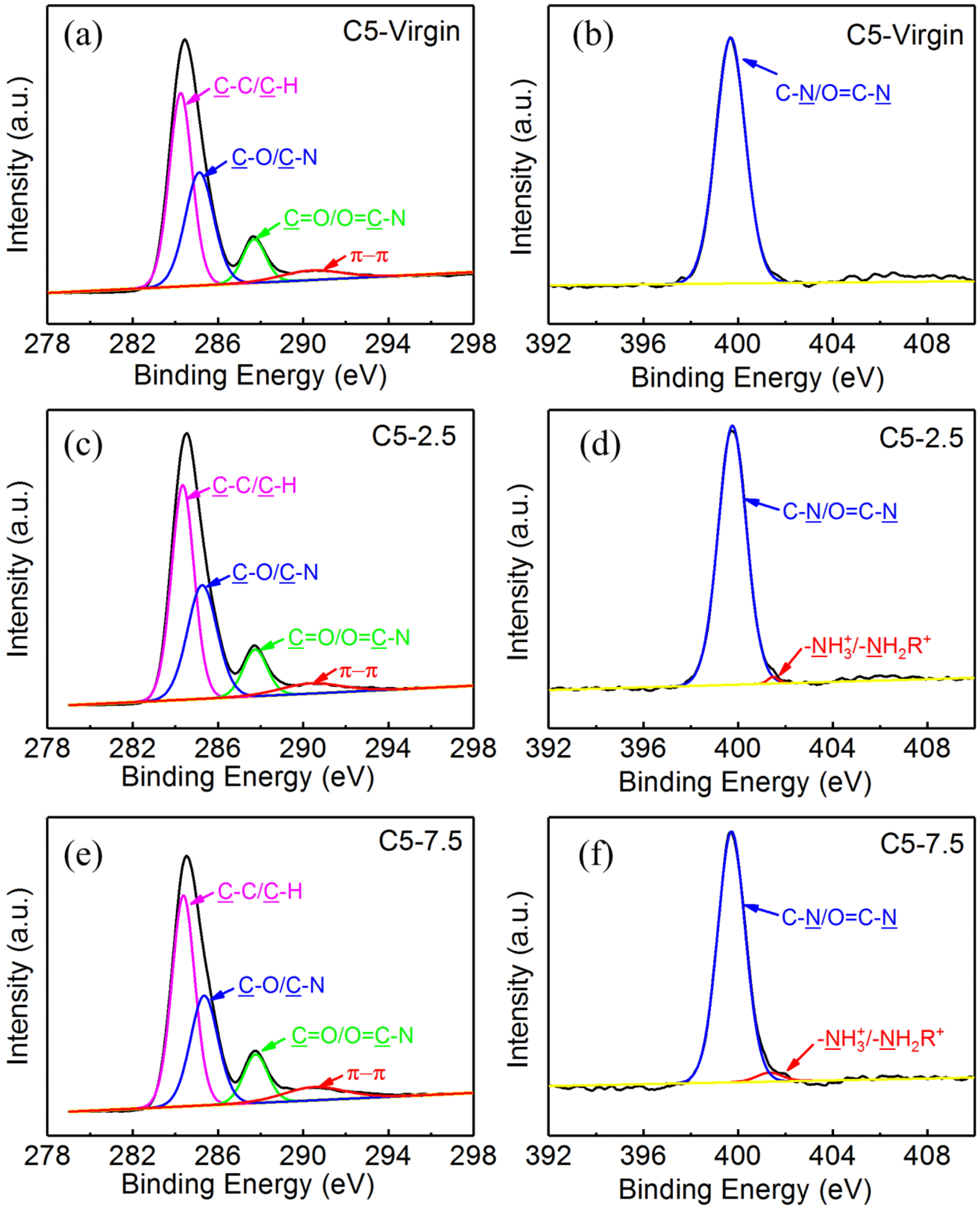

Figure S4. The C1s spectra and N1s spectra of C5 membranes: (a, b) prior to exposure and (c, d, e, f) after 6 months of exposure to the acid solutions.

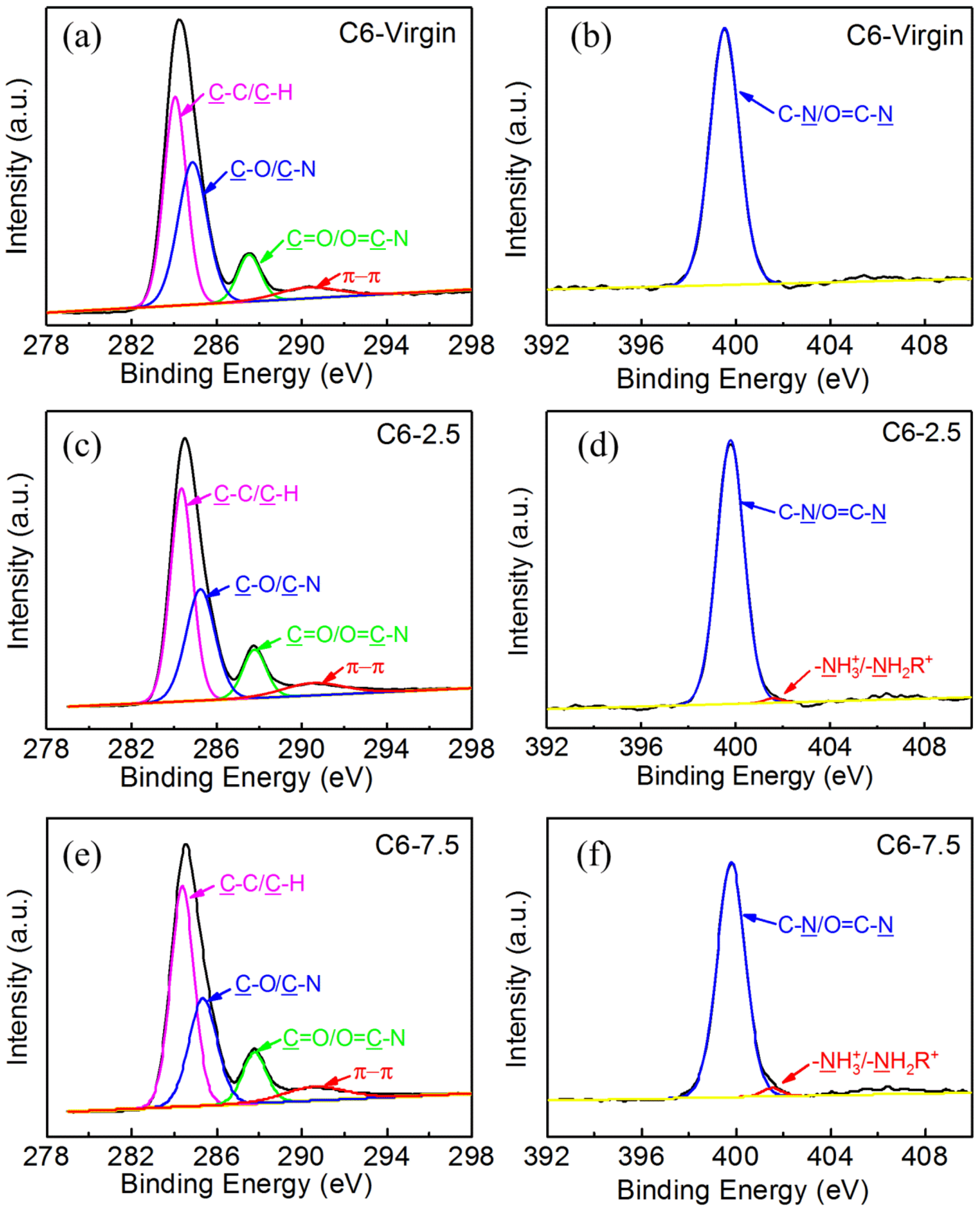

Figure S5. The C1s spectra and N1s spectra of C6 membranes: (a, b) prior to exposure and (c, d, e, f) after 6 months of exposure to the acid solutions.
